# An ArsR/SmtB family member regulates arsenic resistance genes unusually arranged in *Thermus thermophilus* HB27

**DOI:** 10.1111/1751-7915.12761

**Published:** 2017-07-11

**Authors:** Immacolata Antonucci, Giovanni Gallo, Danila Limauro, Patrizia Contursi, Ana Luisa Ribeiro, Alba Blesa, José Berenguer, Simonetta Bartolucci, Gabriella Fiorentino

**Affiliations:** ^1^ Dipartimento di Biologia Università degli Studi di Napoli Federico II Complesso Universitario Monte S. Angelo 80126 Naples Italy; ^2^ Centro de Biología Molecular Severo Ochoa (CSIC‐UAM) Campus Universidad Autónoma de Madrid 28049 Madrid Spain

## Abstract

Arsenic resistance is commonly clustered in *ars* operons in bacteria; main *ars* operon components encode an arsenate reductase, a membrane extrusion protein, and an As‐sensitive transcription factor. In the As‐resistant thermophile *Thermus thermophilus* HB27, genes encoding homologues of these proteins are interspersed in the chromosome. In this article, we show that two adjacent genes, *TtsmtB,* encoding an ArsR/SmtB transcriptional repressor and, *TTC0354,* encoding a Zn^2+^/Cd^2+^‐dependent membrane ATPase are involved in As resistance; differently from characterized *ars* operons, the two genes are transcribed from dedicated promoters upstream of their respective genes, whose expression is differentially regulated at transcriptional level. Mutants defective in *TtsmtB* or *TTC0354* are more sensitive to As than the wild type, proving their role in arsenic resistance. Recombinant dimeric *Tt*SmtB binds *in vitro* to both promoters, but its binding capability decreases upon interaction with arsenate and, less efficiently, with arsenite*. In vivo* and *in vitro* experiments also demonstrate that the arsenate reductase (*Tt*ArsC) is subjected to regulation by *Tt*SmtB. We propose a model for the regulation of As resistance in *T. thermophilus* in which *Tt*SmtB is the arsenate sensor responsible for the induction of *Tt*ArsC which generates arsenite exported by TTC0354 efflux protein to detoxify cells.

## Introduction

Arsenic (As) is an ubiquitous metalloid naturally present in soil, water and air that adversely affects human and animal health. Because of its abundance and toxicity, monitoring arsenic concentration in the environment and in several foodstuffs used for human consumption is very important.

Under reducing conditions, the highly toxic trivalent arsenite, As(III), is the more abundant form, whereas in oxygenated environments, the less‐toxic and more stable pentavalent arsenate, As(V), dominates. Arsenite enters the cell through aquaglyceroporins; as it has a high affinity for sulfur, it exerts its toxicity through binding to dithiols in proteins, in glutathione (GSH) and in lipoic acid contributing to protein/enzyme inactivation (Liu *et al*., 2004; Meng *et al*., 2004). On the other hand, arsenate enters the cells through phosphate transporters and its toxicity is mediated by replacing phosphate in essential biochemical reactions (Tawfik and Viola, 2011; Kamerlin *et al*., 2013).

The abundance of arsenic in the environment has guided the evolution of multiple defence strategies in almost all microorganisms (Contursi *et al*., 2013); for instance, despite being toxic, some microorganisms also use arsenic as electron acceptor in anaerobic respiratory chains or as electron donor for chemolythotrophic growth and even for anoxigenic photosynthesis (Kulp *et al*., 2008; van Lis *et al*., 2013); other microorganisms are able to methylate inorganic arsenic or de‐methylate the organic forms (Qin *et al*., 2006).

In general, in many prokaryotes, arsenic resistance is linked to the presence of plasmid‐ or chromosomally encoded *ars* operons with a variable number of genes. The simplest resistance system involves cytoplasmic reduction of As(V) to As(III) by arsenate reductase and further extrusion of As(III) by a membrane protein whose expression is regulated by ArsR, a trans‐acting repressor of the ArsR/SmtB family (Wysocki *et al*., 2003; Jacobson *et al*., 2012). Arsenate reductases use thioredoxin, glutaredoxin or mycoredoxin as electron donors (Rosen, 2002), whereas arsenite extrusion is mediated by two families of proteins: ArsB proteins, that have been found only in bacteria (Rosen, 1999), and Acr3 proteins, with representatives in bacteria, fungi and plants (Indriolo *et al*., 2010). Two additional genes encoding an ATPase component (ArsA) of the arsenite transporter (ArsB) and a metallochaperon (ArsD) can increase the efficiency of the arsenite efflux system (Lin *et al*., 2006) in prokaryotes. Genomic analysis on thousands of microorganisms revealed new genes in *ars* operons with unknown functions. As an example, parallel pathways for organic arsenicals have been recently identified (Yang and Rosen, 2016).

Regarding transcriptional regulators, different families of metal‐sensing proteins (identified as family HTH_5 in the Pfam database) have been described in bacteria, with ArsR/SmtB being the most extensively studied and named after its founding members, *Escherichia coli* ArsR and *Synechococcus* PCC 7942 SmtB (Wu and Rosen, 1991; Morby *et al*., 1993). The members of the ArsR/SmtB family have many common features, but also display a great diversity in metal‐sensing motifs and metal‐binding mechanisms. They share a dimeric structure and contain a helix–turn–helix (HTH) or winged HTH DNA‐binding domain, and their sequence includes an ELCV(C/G)D motif, defined as the metal‐binding box, located within the HTH region (Shi *et al*., 1994; Cook *et al*., 1998). Binding of a metal to this motif interferes with DNA binding. The SmtB protein binds to imperfect 12‐2‐12 inverted repeats (or a half of this site) located within the operator–promoter region or overlapping the transcriptional start site of the regulated promoters. As binding to the metal alleviates transcriptional repression, these factors act as metal‐sensitive transcription repressors (Osman and Cavet, 2010). Despite conservation of the metal‐binding motif, the selectivity for the metal and its binding mode differ among SmtB homologues due to the capability of the conserved Cys residues to form metal‐thiolate bonds of different geometry and coordination (Guerra and Giedroc, 2012). This is consistent with the hypothesis that metal‐binding sites in DNA‐binding proteins have evolved convergently in response to environmental pressures (Ordonez *et al*., 2008).

With all of the activities above described, microbes actively participate in the geochemical cycling of arsenic in the environment, promoting or inhibiting arsenic release (Fernandez *et al*., 2014). Specifically, thermophilic microorganisms influence the biogeochemistry of arsenic compounds in different geothermal springs (Donahoe‐Christiansen *et al*., 2004). However, to date, information regarding the molecular mechanisms of arsenic resistance in these habitats is still preliminary although it is an essential prerequisite to develop effective systems for arsenic sensing and monitoring in the environment.

The thermophilic Gram‐negative bacterium *Thermus thermophilus* HB27 is capable of growing in the presence of arsenate and arsenite in concentrations that are lethal for other microorganisms. The putative resistance genes have not been found in a single resistance operon but scattered and associated with chromosomal genes apparently not functionally related. In a recent work, we demonstrated the involvement of a thioredoxin‐coupled arsenate reductase (*Tt*ArsC) in the As‐resistance mechanism and hypothesized that arsenic‐dependent induction of *TtarsC* could be mediated by factors such as ArsR/SmtB transcriptional regulators (Del Giudice *et al*., 2013).

In this study, we employ a genetic and biochemical approach to demonstrate that two adjacent genes, *TTC0353* (*TtsmtB*), encoding a putative ArsR/SmtB transcriptional repressor, and *TTC0354*, encoding a recently described membrane Zn^2+^/Cd^2+^ ATPase (Schurig‐Briccio and Gennis, 2012), play a key role in the arsenic resistance.

## Results

### TtsmtB and TTC0354 are arsenic‐regulated genes

The *TTC0353* gene of *T. thermophilus* HB27 encodes a putative protein annotated as a member of the ArsR/SmtB family of transcriptional regulators herein named *Tt*SmtB. *Tt*SmtB is a 123‐amino‐acid‐long protein (predicted molecular weight of 13 508.79 Da and a pI of 8.54) with a HTH DNA‐binding motif and a conserved ELCVCD metal‐binding box located in the α‐3/α‐4 helices and in the α‐4 helix respectively (Fig. S1A). Sequence alignment showed 50% of identity with the structurally characterized SmtB transcriptional repressor from *Synechococcus* PCC 7942 (VanZile *et al*., 2000). Secondary and tertiary structure predictions (using the software I‐TASSER at http://zhanglab.ccmb.med.umich.edu/I-TASSER/) revealed an organization in six α‐helices and one β‐sheet comparable to that found in ArsR/SmtB regulators (Cook *et al*., 1998; Fig. S1B). Moreover, the presence of the conserved metal‐binding box and of a cysteine residue (Cys 10) at the N‐terminus putatively involved in metal binding strongly suggests a key role for this protein in metal sensing.

*TTC0354* is separated from *TtsmtB* by 32 bp and encodes a putative membrane metal transporter (predicted molecular weight of 71 814.95 Da and a pI of 8.21) with a heavy‐metal‐associated (HMA) motif displaying ATPase activity stimulated by cations, whose role in metal tolerance is not clear (Schurig‐Briccio and Gennis, 2012; Fig. S1C).

**Figure 1:**
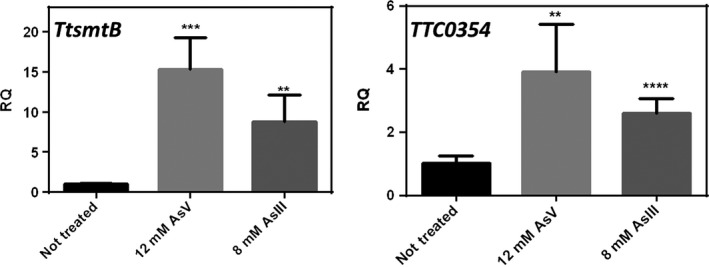
Differential induction of *TtsmtB* and *TTC0354*. qRT‐PCR expression analysis of *TtsmtB* (*TTC0353*) and *TTC0354*, from *T. thermophilus* HB27 exponential cultures grown for 45 min with arsenate (12 mM) or arsenite (8 mM). Error bars indicate the standard deviation of the average values in two independent experiments in triplicate samples (**P < 0.01; ***P < 0.001; ****P < 0.0001).

To analyse whether the expression *of TtsmtB* and *TTC0354* was regulated by arsenic, qRT‐PCR assays were carried out on RNAs isolated from cells treated with arsenate or arsenite at subinhibitory concentrations. As shown in Fig. 1, both ions determine significant increase in the transcription of *TtsmtB* (15‐fold and 9‐fold) and *TTC0354* (4‐fold and 3‐fold).

### Identification of the promoters of the arsenic‐related genes

**Figure 2:**
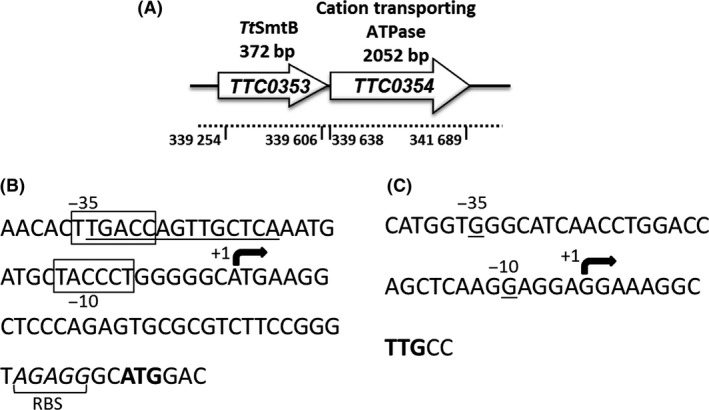
Transcriptional start site determination of *TTC0354* and *TtsmtB*. (A). Genomic context of *TtsmtB* and *TTC0354*. (B). *TTC0354* promoter sequence analysis. The mapped transcription start site (+1) is marked with an arrow. −10 and −35 regions from the transcription start site are boxed. Predicted SmtB‐binding site is underlined. The translation start codon is indicated in bold. RBS is marked. (C). Sequence of *TtsmtB* promoter region. The mapped transcription start site (+1) is marked with an arrow. −10 and −35 nt from the transcription start site are underlined. The translation start codon is indicated in bold.

To better understand the function and the regulation of *TtsmtB* and *TTC0354*, we first verified whether the two genes were transcribed from independent promoters (Fig. 2A). For this, we identified transcription start site(s) (TSS) by primer extension. A TSS was detected for *TTC0354* on total RNA isolated from cells grown without As and upstream from this TSS, −10 and −35 boxes could be identified. Interestingly, this region contained a palindromic sequence (6‐2‐6) matching the consensus of the ArsR/SmtB‐binding site (Osman and Cavet, 2010); a putative ATG start codon 38 bp downstream and a conserved ribosome‐binding site (RBS) (−7 from this ATG) were also identified (Fig. 2B).

On the other hand, a TSS could be also identified for *TtsmtB* at position – 3 from the first putative translated nucleotide (TTG) when cells were treated with arsenic, suggesting low expression levels under basal conditions. Furthermore, consensus‐like promoter boxes were not found at the appropriate upstream distance from this TSS probably due to the fact that this promoter is intragenic (Fig. 2C); in fact, *TtsmtB* gene overlaps with an upstream gene (*TTC0352*) encoding the putative small subunit of pyridoxal 5′‐phosphate (PLP) synthase, an enzyme for *de novo* biosynthesis of PLP coenzyme, with no obvious functional relation to As resistance.

### Role of TtSmtB and TTC0354 in arsenic resistance

As the aim of our study was to establish a role for *Tt*SmtB and TTC0354 in the arsenic resistance, the genes were inactivated by insertion of a gene cassette encoding a thermostable resistance to kanamycin in *TtsmtB* (Fig. S2) or the suicide vector pK18 in *TTC0354* (Fig. S3). The screening of kanamycin‐resistant recombinants was carried out by PCR, and the deletion/insertion was confirmed by sequence analysis.

In the absence of arsenic, mutant and wild‐type strains grew at similar rates, showing that the genes were not relevant for *T. thermophilus* viability under such conditions. Nevertheless, when they were phenotypically analysed by MIC assays, a decrease in arsenate and arsenite resistance in both mutants was observed (Table 1). Inactivation of *TtsmtB* resulted in 2.5‐fold and 1.5‐fold reductions in the MIC of arsenate and arsenite, respectively, while mutation of *TTC0354* leads to 15‐fold and 13‐fold reduction respectively. These results indicate that the two genes are relevant for arsenic resistance and are consistent with the predicted functions as an arsenic transcriptional regulator and an efflux transporter respectively.

**Table 1:** Bacterial resistance to arsenic

Strain	MIC	
	As(V)	As(III)
*Thermus thermophilus* HB27	44 mM	40 mM
*Thermus thermophilus ΔsmtB::kat*	18 mM	32 mM
*Thermus thermophilus TTC0354::pK18*	3 mM	3 mM

### Purification and structural characterization of TtSmtB

To better characterize the regulatory role of *Tt*SmtB, the corresponding gene was cloned in pET28b (+) plasmid to generate a His‐tagged fusion protein that was expressed in *E. coli* BL21‐CodonPlus(DE3)‐RIL and purified to homogeneity.

Far‐UV circular dichroism spectra showed a typical circular dichrogram of a helical protein with negative maxima at 208 and 222 nm and one positive peak at 195 nm (Fig. 5A), indicative of a predominantly folded structure with an α‐β content. Data obtained from deconvolution of the CD spectra established that the recombinant protein was properly folded with approximately 32.6% α‐helix and 37.1% turn sheet, according to the structural model (Fig. S1). Furthermore, gel filtration experiments showed that the protein is a homodimer of about 27 kDa in solution.

As *Tt*SmtB is a dimer with three cysteine residues (Cys 10, Cys 62 and Cys 64) per subunit, we analysed whether they were involved in intramolecular disulfide bridges or not by treating the protein with iodoacetamide, followed by trypsin (and chymotrypsin) digestion, and MALDI‐TOF mass spectrometry peptide analysis (70.5% sequence coverage). The mass of peptides containing the cysteines was increased of 57 Da indicating that such residues are not involved in disulfide bridges and suggesting that they are likely involved in metal coordination.

### Binding analysis of TtSmtB to sequences upstream of TtsmtB, TTC0354 and TtarsC

**Figure 3:**
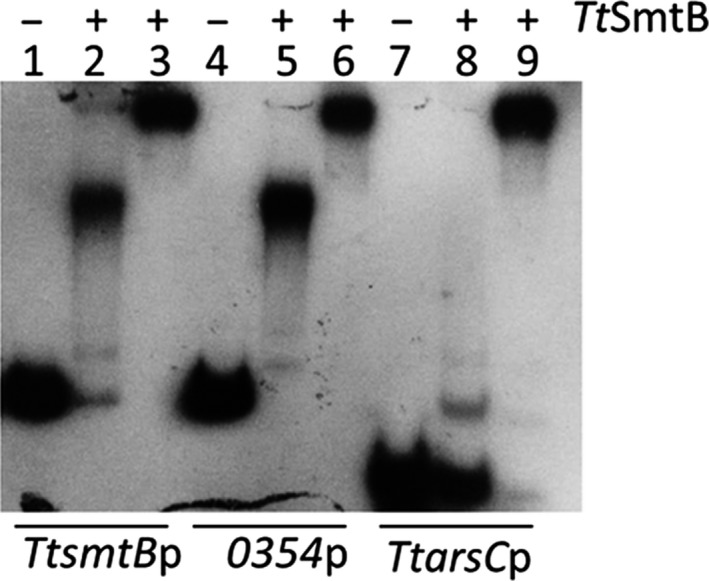
Activity of *Tt*SmtB. Binding of 2.5 μM (lanes 2, 5, 8) and 7.5 μM (lanes 3, 6, 9) *Tt*SmtB to *TtsmtB*p, *0354*p and *TtarsC*p.

To verify whether *Tt*SmtB recognizes the identified regulatory regions, we performed EMSAs. We tested the following: (i) *TtsmtB* promoter to verify autoregulation; (ii) *TTC0354* promoter, the only containing a palindromic sequence matching the consensus of the ArsR/SmtB‐binding site in other organisms; (iii) the region upstream of *TtarsC* (*TTC1502)*, to examine whether arsenate reductase could be a regulatory target of *Tt*SmtB (Del Giudice *et al*., 2013). All the DNA fragments, obtained through PCR amplifying 149, 143 and 78 bp regions upstream of *TtsmtB, TTC0354* and *TtarsC,* respectively, were incubated with 2.5 and 7.5 μM of purified recombinant protein. The results show that *Tt*SmtB can form complexes with different mobility and intensity in the three promoters in a concentration‐dependent manner, suggesting that the protein binds to them differentially (Fig. 3).

Further analyses were performed to better characterize the interaction of *Tt*SmtB with the putative promoter controlling *TTC0354* (*0354*p) as well as its own promoter (*TtsmtB*p) (Fig. S4). We first assessed whether *Tt*SmtB binding to the promoters was specific; the *0354*p‐*Tt*SmtB complexes dissociated in the presence of an excess of unlabelled specific probe whereas remained bound in the presence of a molar excess (200× and 400×) of unspecific DNA. Titration with increasing concentrations of *Tt*SmtB indicated that the protein binds to this region in a concentration‐dependent manner; at saturating concentrations, the protein determined a shift with low mobility, suggesting either that other binding sites with different affinities could exist in the DNA sequence analysed or that multiple dimers could associate with the cognate DNA (Kar *et al*., 2001). The profile obtained by fitting densitometric data to a binding curve with a Hill slope gave an overall apparent equilibrium dissociation constant (*K*_d_) of 1.4 ± 0.07 μM, suggesting that DNA binding is cooperative, as reported for other characterized SmtB family members (Mandal *et al*., 2007; Chauhan *et al*., 2009). On the other hand, titration of *Tt*SmtB showed that the protein also forms multiple complexes with its own promoter and binds cooperatively but with lower affinity (*K*_d_ of 5.0 ± 0.3 μM) and lower specificity (Fig. S4).

**Figure 4:**
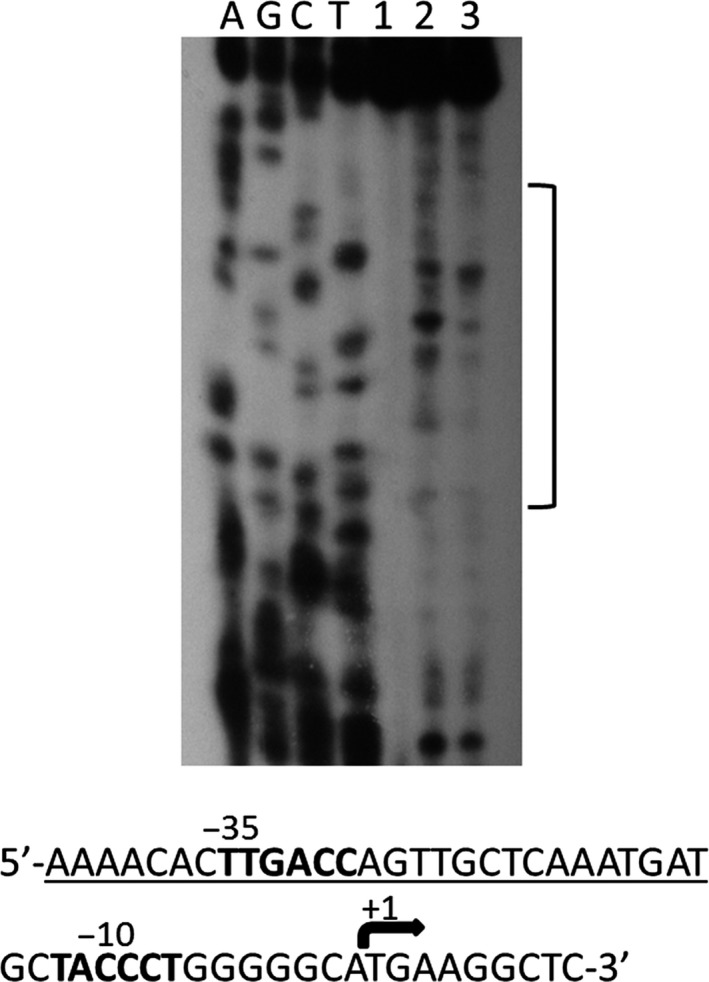
DNase I footprinting analysis of *Tt*SmtB on *TTC0354* promoter. Footprinting was performed at the non‐template strand using 0.0 μg (lanes 1–2) or 4 μg (∼3 μM, lane 3) of purified *Tt*SmtB, in the absence (lane 1) or in the presence of 3 U of DNase I (lanes 2–3). DNA fragments were analysed in parallel with a sequencing reaction by denaturing gel electrophoresis. Position of the footprint (sequence underlined) relative to the *TTC0354* promoter is shown.

To map the *Tt*SmtB‐binding site on the *TTC0354* promoter, DNase I footprinting was performed using a 143 bp DNA including sequences −100 to +42 from the mapped transcription start site. As shown in Fig. 4, a 28 bp protected region was observed containing the TTGCTCAA sequence matching a consensus with binding sites of ArsR/SmtB members. Interestingly, this sequence overlaps to −35 box, strongly suggesting that *Tt*SmtB binding at this promoter could hamper RNA polymerase binding.

### TtSmtB *in vitro* interaction with arsenic

The subsequent task was to determine whether *Tt*SmtB binds arsenic *in vitro* and whether the interaction modifies its DNA‐binding ability.

**Figure 5:**
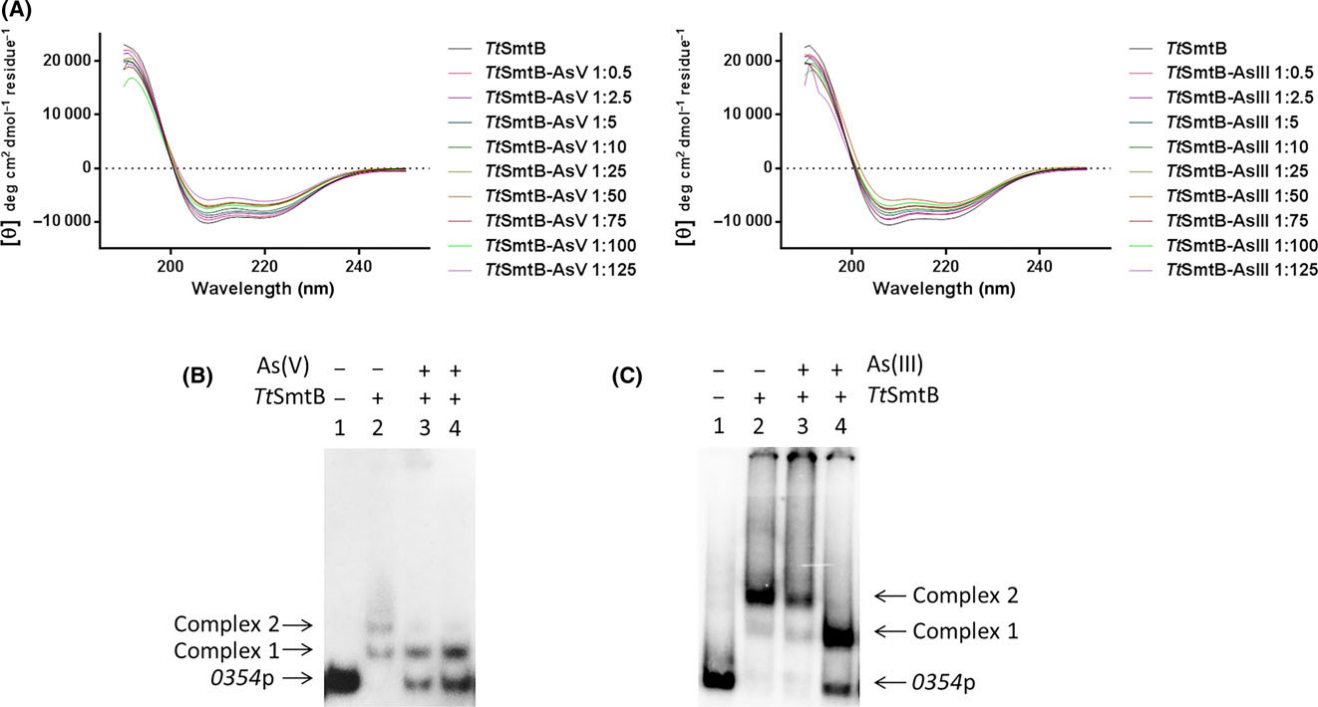
*Tt*SmtB interaction with arsenic. (A). Far‐UV CD spectrum of *Tt*SmtB with increasing amounts of As(V) and As(III). (B). Binding of *Tt*SmtB to *0354p* without (lane 2) and with arsenate at molar ratio of 1:50 and 1:100 (lanes 3–4). (C). Binding of *Tt*SmtB to *0354p* without (lane 2) and with arsenite at molar ratio of 1:50 and 1:100 (lane 3–4).

Circular dichroism was employed to investigate the secondary structure of *Tt*SmtB in the presence or absence of increasing concentrations of arsenate and arsenite. The results in Fig. 5A show that the absolute values of the negative peaks at 208 and 220 nm, that are related to the α‐helical content in the protein structure, were progressively increasing at higher arsenate and arsenite concentrations revealing that *in vitro* As determines changes in *Tt*SmtB secondary structure. In more detail, the values of the molar ellipticity per residue at 208 nm, plotted against arsenate and arsenite concentrations, gave binding curves with an apparent equilibrium dissociation constant *K*_d_ of approximately 0.06 and 0.25 mM respectively (not shown). These values so diverse suggest that arsenate and arsenite cause structural rearrangements with different effect on *Tt*SmtB activity.

Finally, to analyse whether arsenic influences *Tt*SmtB DNA‐binding ability, we performed EMSAs in which DNA‐*Tt*SmtB complex formation was tested after pre‐incubation of the protein with arsenate and arsenite each at 50‐fold and 100‐fold molar excess. The results reported in Fig. 5B and C demonstrate that the interaction of arsenate and arsenite with *Tt*SmtB hampers binding to *0354*p although at different concentrations; in fact, in the presence of arsenate, complex 2 is disrupted at lower concentration than when arsenite is used (Fig. 5B and C lanes 3–4).

### TtSmtB is a repressor *in vivo*

**Figure 6:**
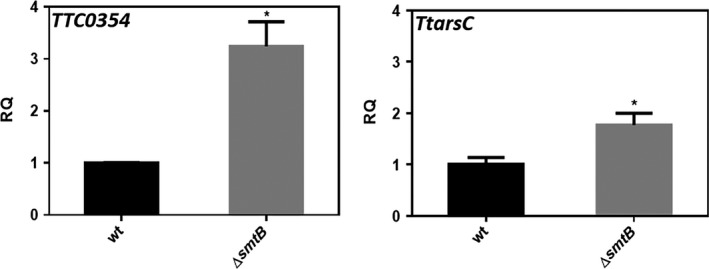
qRT‐PCR analysis of *TTC0354* and *TtarsC* in *T. thermophilus* HB27 (wt) and Δ*smtB* grown without As. The error bars indicate the standard deviation of the average values of two independent experiments in triplicate samples (*P < 0.05).

To confirm the *in vivo* role of *Tt*SmtB as a repressor, we used qRT‐PCR to evaluate in the *ΔsmtB* mutant the expression of the arsenic resistance genes *TTC0354* and *TtarsC* (Del Giudice *et al*., 2013). As shown in Fig. 6, 3‐fold and 2‐fold increases in *TTC0354* and *TtarsC* expressions, respectively, were detected in *ΔsmtB* compared to the wild‐type strain. These results further suggest that the genes investigated are functional targets of *Tt*SmtB that exerts a negative regulation of their transcription *in vivo*.

## Discussion

Thermophilic microorganisms play important roles in arsenic bioavailability in thermal environments and represent good models either to investigate the molecular mechanisms of response to metal stress (Bartolucci *et al*., 2013) or for the development of robust biosensors for arsenic monitoring (Politi *et al*., 2015; Fernandez *et al*., 2016). In a recent study, we demonstrated that the Gram‐negative hyperthermophilic bacterium *T. thermophilus* HB27 can tolerate high concentrations of arsenic and that the genes putatively involved in arsenic resistance are scattered in the chromosome; in particular, a thermostable arsenate reductase (*Tt*ArsC), an enzyme capable of reducing pentavalent arsenate to trivalent arsenite, was characterized (Del Giudice *et al*., 2013; Politi *et al*., 2016).

In the present study, we demonstrate that *Tt*SmtB, encoded by *TTC0353*, is an ArsR/SmtB transcriptional factor exerting negative regulation on two genes, *TtarsC* and *TTC0354*, involved in arsenic resistance. In the genome annotation, *TTC0353* is adjacent to *TTC0354* encoding an ATPase stimulated by cations, particularly Cu^+^, with a heavy‐metal‐associated domain (Schurig‐Briccio and Gennis, 2012). The genomic localization of *TTC0354* and structure predictions of the encoded protein (Fig. S1C) led us to hypothesize that TTC0354 could function *in vivo* as the arsenite efflux transporter, so we further investigated this point. Inactivation of *TTC0354* was decisive to point out its role in arsenic resistance; in fact, *T. thermophilus TTC0354::pK18* is significantly more sensitive to arsenic treatment.

The *ΔsmtB* mutant strain was also obtained; in this strain, the expression levels of *TTC0354* are higher than in the wild type providing experimental evidence that *Tt*SmtB represses gene transcription *in vivo*. Although the derepression was not particularly strong, the data were statistically significant. Moreover, qRT‐PCR experiments performed on the wild type showed that *TtsmtB* transcription is increased upon arsenate/arsenite treatment, confirming its involvement in the arsenic response. Nevertheless, as judged by primer extension experiments, *TtsmtB* expression under basal conditions turned to be low, congruent with its role as a transcriptional regulator. Therefore, the steep induction of *TtsmtB* transcription upon exposure to arsenate/ite is functional to initially cope with the increase in arsenic concentration and afterwards to restore the negative transcriptional control over the key genes once the arsenic has been cleared out.

To elucidate the *in vitro* role of *Tt*SmtB in arsenic sensing and gene regulation, we produced a recombinant form of the protein and demonstrated that, as other members of its family, it is a dimer in solution and has three cysteine residues in a reduced form available to coordinate arsenic binding. EMSAs showed that *Tt*SmtB can form multiple complexes and binds to the different promoters that we tested with high cooperativity; the different shifted bands might represent different forms of the DNA–protein complex as already proposed for the SmtB protein of *Synechococcus* PCC7942 (Kar *et al*., 2001). According to *in vivo* expression data, cooperativity could guarantee that even small variations in intracellular protein concentration can regulate tunable occupancy of DNA target sequences. *Tt*SmtB more strongly and specifically interacts with *TTC0354* promoter through binding to a sequence, identified through DNase I footprinting, corresponding to a consensus palindromic ArsR/SmtB‐binding site, overlapping putative basal transcription elements; this mode of DNA recognition is another common feature of the ArsR/SmtB protein family (Osman and Cavet, 2010). Sequence alignments showed that *Tt*SmtB has one of the three cysteine residues required to form the As(III)‐binding site at the N‐terminus end outside the metal‐binding box (Fig. S1A), so it is conceivable that the geometry of the metal‐binding site could favour arsenate binding (Shi *et al*., 1994). Circular dichroism spectroscopy experiments indicate that both the arsenic ions determine changes in *Tt*SmtB secondary structure and suggest that the protein binds arsenate more efficiently than arsenite. Furthermore, both arsenate and arsenite are able to trigger *Tt*SmtB release from DNA, suggesting that *in vivo* the transcription of target genes could be repressed under basal conditions and allosterically activated upon increase in intracellular arsenate/ite concentration. Interestingly, arsenate binding by *Tt*SmtB hampers DNA interaction better than arsenite, further indicating that in this system, arsenate could be the actual effector of *Tt*SmtB. To our knowledge, this is the first demonstration of an ArsR protein preferentially interacting with arsenate.

**Figure 7:**
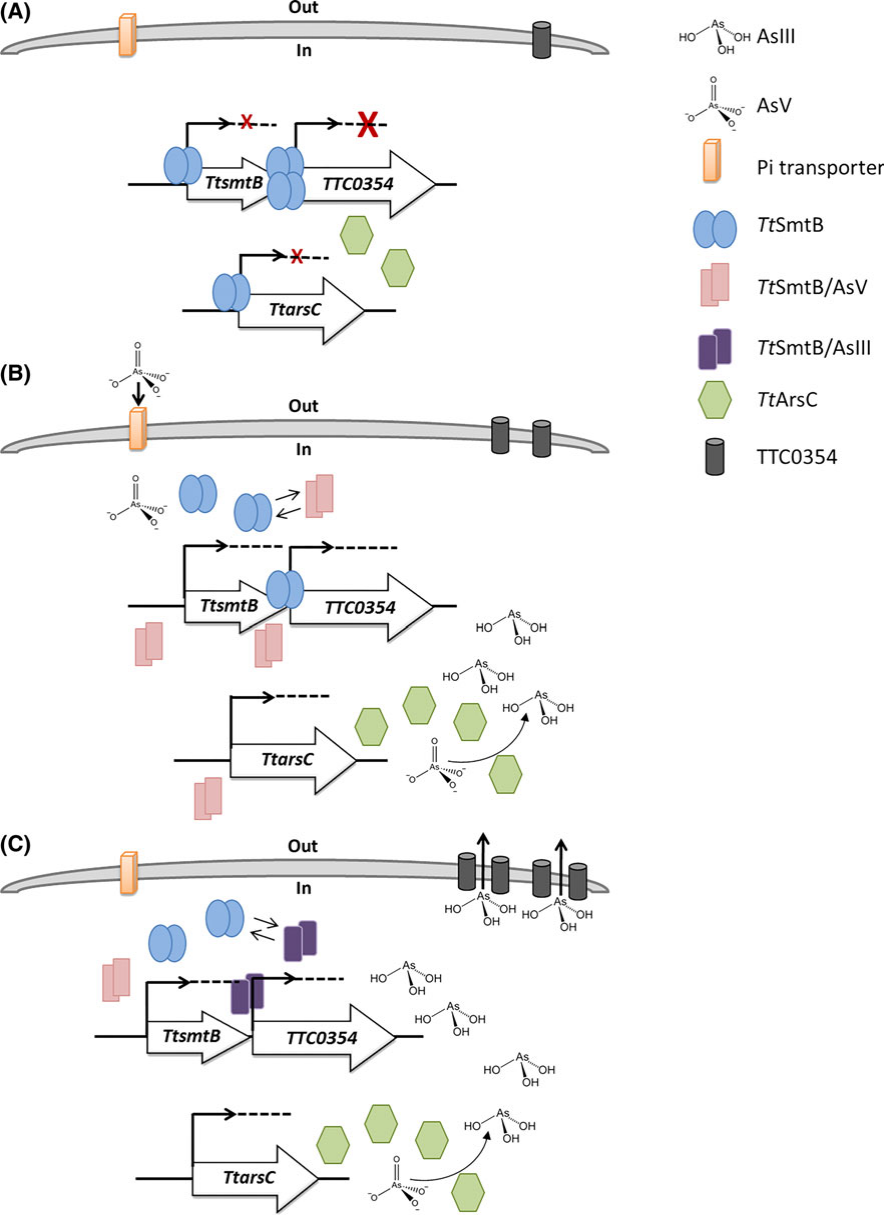
Cartoon of the role of *Tt*SmtB in arsenic resistance. (A). In the absence of arsenic, the dimeric *Tt*SmtB is expressed at low levels and binds target promoters of the indicated genes eliciting transcriptional repression. As demonstrated by *in vitro* DNA‐binding experiments, *0354p* is more tightly repressed. (B). When arsenate enters the cells, it binds to *Tt*SmtB lowering its affinity towards DNA and inducing derepression of *TtsmtB* and *TtarsC*; derepression of *TtarsC* allows a catalytic conversion of arsenate to arsenite. (C). Further intracellular accumulation of arsenite determines complete release of *0354p,* transcriptional activation of the pump and extrusion of arsenite outside the cells. The reduction in intracellular arsenate levels coupled to the efficient export of arsenite restores transcriptional repression.

Based on the results presented here, a model for the role of *Tt*SmtB in As resistance in *T. thermophilus* HB27 is proposed in Fig. 7. Under basal conditions, *Tt*SmtB exerts autoregulation expressing itself at low levels and represses *TTC0354* and *TtarsC* (Fig. 7A). The entry of arsenate into the cells determines structural changes in *Tt*SmtB with the effect of derepressing at first the low‐affinity promoters, with consequent increase in free *Tt*SmtB concentration and in the arsenate reductase *Tt*ArsC (Fig. 7B). Further accumulation of arsenate and arsenite, the latter as product of the *Tt*ArsC‐catalyzed reaction, leads to the full derepression of the TTC0354 efflux pump allowing the efficient transport of arsenite out of the cells (Fig. 7C).

Our findings do not exclude that *TtsmtB/TTC0354/TtArsC* system is the only responsible to cope with arsenic toxicity and suggest that alternative regulatory circuits or protein partners could participate, as already seen in other microorganisms (Wang *et al*., 2004). Moreover, both *Tt*SmtB and TTC0354 might play a broader role in response to metal stress.

Altogether these results give insights into the mechanisms of metal‐regulated gene expression in *T. thermophilus* pointing out to substantial differences with better characterized *ars* systems (Fernandez *et al*., 2016); the role of *Tt*SmtB and TTC0354 and their regulatory sequences in arsenic sensing add a new piece in the puzzle of the molecular machinery of *T. thermophilus* arsenic resistance and represent an important progress both for the development of effective, safe and stable whole‐cell arsenic biosensors and/or for the exploitation of novel bioremediation processes.

## Experimental procedures

### Bacterial strains and culture conditions

*Escherichia coli* strains were grown in Luria Bertani (Miller, 1972) medium at 37 °C with 50 μg ml^−1^ kanamycin and/or 33 μg ml^−1^ chloramphenicol and/or hygromycin B as required. *T. thermophilus* HB27 wild‐type strain (purchased from DSMZ) was grown aerobically at 70 °C in TM medium without or with 8 mM NaAsO_2_ (Sigma, referred to throughout this article as arsenite) or 12 mM KH_2_AsO_4_ (Sigma, referred to throughout this article as arsenate) as described (Del Giudice *et al*., 2013); 8 mM arsenite and 12 mM arsenate were chosen because they correspond to subinhibitory concentration values determined in a previous work (Del Giudice *et al*., 2013). *T. thermophilus ΔsmtB::kat* and *TTC0354::pK18* mutants were grown aerobically at 70 °C in TM medium containing kanamycin (30 μg ml^−1^).

For qRT‐PCR experiments, 50 ml cultures of *T. thermophilus* HB27 and *ΔsmtB::kat* were grown up to 0.5 OD_600 nm_ and harvested at 0 and 45 min after the addition of 8 mM NaAsO_2_ or 12 mM KH_2_AsO_4_ and immediately spun down, and pellets were kept at −80 °C.

For the determination of minimum inhibitory concentration (MIC), exponentially growing cultures were diluted to OD_600_ = 0.08 in 24‐well plates (Corning, New York, USA) in TM medium with increasing concentrations of arsenic (0–50 mM arsenate or 0–45 mM arsenite) as described in the Manual of Antimicrobial Susceptibility Testing (Coyle, 2005) and grown at 70 °C for 18 h; for each determination, two independent experiments with triplicate samples were carried out. Minimum inhibitory concentration (MIC) was determined as the lowest concentration of arsenic that completely inhibited the growth of the strain as evaluated by OD_600 nm_ after incubation for 18 h under optimal conditions. MIC definition is different from that previously described (Del Giudice *et al*., 2013).

Strain genotypes and sources are summarized in Table S1.

### DNA and RNA extraction

*Thermus thermophilus* HB27 genomic DNA was prepared following reported procedures (Pedone *et al*., 2014). Total RNA was extracted using the RNeasy Mini Kit (Qiagen, Hilden, Germany). The extracted RNA samples (20 μg) were then diluted to 0.2 mg ml^−1^ for DNase I treatment with the Ambion^®^ TURBO™ DNase according to the manufacturer's instructions.

### qRT‐PCR

To determine whether the expression *of TtsmtB* and *TTC0354* genes was induced by arsenic, and to verify the expression of *TTC0354* and *TTC1502* (*TtArsC*) in the *ΔsmtB* strain, qRT‐PCRs were performed using the StepONEPlus Real‐Time PCR system (Applied Biosystems, Foster City, California, USA) and the SYBR Select Master Mix kit (Applied Biosystems). Total RNA extracted from *T. thermophilus* HB27 and *ΔsmtB* cells was digested with TURBO DNase, RNase‐free (Contursi *et al*., 2010). The cDNAs were synthetized using a mixture of the corresponding reverse primer (*0353rv*,* 0354realrv* or *arsCrealrv*) and the 16S reverse primer (*16Sthrv*), used as internal control for normalization. The specific cDNAs synthesized were amplified using the following primers: *smtBrealfw* and *0353rv*;* 0354realfw* and *0354realrv*;* arsCrealfw* and *arsCrealrv;* or *16Sthfw* and *16Sthrv* (Table S2) designed using Primer Express 2.0 software (ABI Biosystems), and amplified 107 bp, 89 bp and 100 bp specific products of *TtsmtB*,* TTC0354* and *TtarsC*, respectively. Reaction optimization was assessed for each template to generate standard curves and calculate the amplification efficiency.

For the amplification of the specific cDNAs, 25 ng from the RT‐reaction mixture was used, whereas 0.002 ng was used to amplify the 16S fragment. DNA contamination was tested by the inclusion of a control without reverse transcriptase for each RNA sample. Two independent experiments were performed, and each sample was always tested in triplicate. The amplification data were analysed using the StepONE software (Applied Biosystems), and induction folds were calculated by the comparative Ct method. The relative expression ratio of the target gene, *TtsmtB* or *TTC0354*, versus that of the *16S* rRNA gene was calculated as described (Pfaffl, 2001).

### Primer extension analysis of transcription start site

To determine the first transcribed nucleotides of *TtsmtB* and *TTC0354*, total RNA extracted from *T. thermophilus* HB27 cells was subjected to primer extension analysis as described (Fiorentino *et al*., 2011), using the primers *0353pr(ext) rv 2 and 0354rv* (Table S2). The same primers were used to produce a sequence ladder using the Thermo Sequenase Cycle Sequencing Kit (Affymetrix, Santa Clara, California, USA) according to the manufacturer's instructions to locate the products on 6% urea polyacrylamide gels.

### Cloning, expression and purification of TtSmtB

The gene encoding *Tt*SmtB was amplified by PCR from *T. thermophilus* HB27 genomic DNA, using Taq DNA polymerase (ThermoFisher Scientific, Waltham, Massachusetts, USA) and the primers containing the NdeI (*smtBfw*) and HindIII (*smtBrv*) sites at the 5′ and 3′ ends respectively. Amplified fragments were purified, digested and cloned into NdeI/HindIII‐digested pET28b(+) vector (Novagen). For protein expression, *E. coli* BL21‐CodonPlus (DE3)‐RIL cells transformed with pET28*/TtsmtB* were grown in LB medium containing kanamycin (50 μg ml^−1^), chloramphenicol (33 μg ml^−1^) and 0.25 mM ZnSO_4_. When the culture reached 0.7 OD_600 nm_, protein expression was induced by the addition of 0.5 mM isopropyl‐1‐thio‐β‐D‐galactopyranoside (IPTG) and the bacterial culture was grown for 16 h at 22 °C. Cells were harvested and lysed by sonication in 50 mM Tris–HCl, pH 7, as described before (Fiorentino *et al*., 2014). The recombinant protein was purified to homogeneity through a heat treatment of the cell extract (65 °C for 10 min) followed by HiTrap Heparin chromatography (5 ml; GE Healthcare) connected to an AKTA Explorer system (GE Healthcare). The fractions containing His‐*Tt*SmtB were pooled, concentrated by ultrafiltration and dialysed for 16 h at 4 °C against 50 mM Tris–HCl, pH 7.0, and 0.15 M NaCl. To prevent protease activity, an inhibitor cocktail (Roche) was added at each step. The histidine tag was removed using 10 U of thrombin (Sigma, St. Louis, Missouri, USA) for 1 mg of His‐*Tt*SmtB yielding *Tt*SmtB, a protein of 13.5 kDa. The purified proteins were stored in aliquots at −20 °C.

### TtSmtB structural characterization

To determine the quaternary structure of *Tt*SmtB, the native molecular mass was determined by loading the purified protein to an analytical Superdex PC75 column (0.3 × 3.2 cm) in 50 mM Tris–HCl, pH 7, and 0.2 M NaCl. The column was calibrated using a set of gel filtration markers (low range; GE Healthcare), including bovine serum albumin (67.0 kDa), ovalbumin (43.0 kDa), chymotrypsinogen A (25.0 kDa) and RNase A (13.7 kDa).

Determination of putative disulfide bonds was obtained by mass spectrometry through MALDI mapping after *Tt*SmtB carbamidomethylation with iodoacetamide, and trypsin and chymotrypsin digestion to detect carbamidomethyl‐cysteine‐containing peptides as described (Moinier *et al*., 2014).

Analysis of secondary structure was performed by registering far‐UV circular dichroism spectra in a Jasco J‐815 CD spectrometer, equipped with a Peltier‐type temperature control system (PTC‐423S/15 model) using protein concentration of about 3 μM in a 25 mM Na‐phosphate, pH 7.0 buffer. CD spectra were recorded as described (Prato *et al*., 2008). Spectra were analysed for secondary structure amount according to the Selcon method using Dichroweb (Whitmore and Wallace, 2008). CD spectra were also registered to titrate *Tt*SmtB with increasing amounts of arsenate and arsenite: 6, 30, 60, 118, 230, 590, 890, 1200, 1500 μM. CD titration curves (obtained in triplicate) were fitted in Prism 6.0 using the equation for one binding site to determine the dissociation constants.

### Electrophoretic mobility shift assay

To determine the binding of *Tt*SmtB to the putative promoter regions of *TtsmtB, TTC0354* and *TtarsC*, electrophoretic mobility shift assays (EMSAs) were performed. The promoter regions were amplified by PCR using specific primer pairs (one radiolabelled with γ^32^P dATP and polynucleotide kinase): *0353pr(ext)fw* and *0353pr(ext)rv2*,* 0354footprint fw* and *0354footprint rv, ArsCprfw* and *ArsCprrv* (Table S2) giving 149, 143 and 78 bp fragments respectively. EMSA reactions were set up as described (Fiorentino *et al*., 2007), using 2.5 or 7.5 μΜ of proteins. Sequence‐specific binding to *0354*p was evaluated in a competition assay using competitors at molar ratio of 1:200 and 1:400. As aspecific DNA, a 150 bp coding region from *Sulfolobus solfataricus* was amplified with *VP2 fw* and *VP2 rv* primers (Fusco *et al*., 2013). To quantify the interaction between *Tt*SmtB/*TtsmtB*p and *Tt*SmtB/*0354*p, the DNA was incubated with increasing amounts of the protein (0–15 μΜ); the complexes were separated, and the gels were processed and analysed by phosphor imaging using Quantity One software (Bio‐Rad) as already described (Fiorentino *et al*., 2011).

To determine whether arsenite and arsenate were ligands for *Tt*SmtB and evaluate their possible effect on binding to the target *0354*p, 2.5 μΜ of protein was pre‐incubated with NaAsO_2_ or KH_2_AsO_4_ at molar ratio of 1:50 and 1:100 (considering *Tt*SmtB as a dimer).

### DNase I footprinting

A probe containing the promoter region of *TTC0354* was produced by PCR using a combination of *0354footprint fw* and *0354footprint rv*; the latter was 5′ end labelled with T4 polynucleotide kinase and [γ‐^32^P] ATP. The labelled PCR product of 143 bp (about 40 nM) was incubated with 4 μg of pure *Tt*SmtB in binding buffer and digested with three units of DNase I (Roche) for 1 min at 37 °C. Subsequent steps were performed as described (Fiorentino *et al*., 2011). Labelled primer was as also used to generate a dideoxynucleotide sequence ladder with Thermo Sequenase Cycle Sequencing Kit (Affymetrix) using 0.1 pmol of the same PCR fragment as the template and following the manufacturer's instructions.

### Construction of *T. thermophilus* mutants

To obtain a *ΔsmtB::kat* deletion mutant of *T. thermophilus* HB27, the chromosomal *TtsmtB* gene was replaced with the kanamycin nucleotidyltrasferase gene (*kat*) cassette by double homologous recombination. Two regions upstream and downstream of *TtsmtB* (arm UP and arm DW) were amplified by PCR using *T. thermophilus* HB27 genomic DNA as template. For arm UP, the forward primer (UP fw SmtB *Eco*RI) and the reverse primer (New UP rv SmtB *Xba*I) contained EcoRI and XbaI sites respectively (Table S2). For arm DW, the forward primer (New DW fw SmtB *Xba*I) and the reverse primer (DW rv SmtB *Hind*III) contained XbaI and HindIII sites. The resulting products (909 bp arm UP and 1014 bp arm DW) were digested, purified, ligated (in 1:1 molar ratio) and cloned into pUC19, giving the pUC19*ΔsmtB* vector. The *kat* cassette, extracted from pUC19‐*kat* after XbaI digestion, was inserted at the XbaI site of pUC19*ΔsmtB*. The resulting vector was named pUC19*ΔsmtB::kat*. The orientation of *kat* cassette was confirmed by restriction analysis. The pUC19Δ*smtB*::*kat* plasmid was HindIII‐digested and used in linear form to transform *T. thermophilus* HB27 as described above.

The replacement of the *TtsmtB* gene was verified by PCR on the genomic DNA of the transformants; three primer sets (Table S2) were used: one pair (*0351promfw*/*0351promrv*) amplified a region in both deleted and wild‐type strains; another one (*0352fw*/*0353rv*) amplified a region only in the wild‐type strain; the last pair (*smtBfw*/*smtBrv*) amplified a fragment of 1125 bp corresponding to the *kat* gene in the mutant strain compared to the *TtsmtB* gene of 372 bp in the wild type. The *kat* insertion was further confirmed by DNA sequencing.

The *TTC0354* mutant was obtained by following a single‐recombination strategy. For this, a 1407 bp internal fragment of the *TTC0354* gene was amplified with primers *0354Eco* and *0354Hind* and further digested with EcoRI and HindIII restriction enzymes, which targets were included in the primer's sequence (Table S2). The fragment was subsequently cloned into the same sites of suicide vector pK18, conferring thermostable resistance to kanamycin, and the resulting plasmid pK18‐*Δ0354* was used to transform *T. thermophilus* as described (Blesa *et al*., 2017). Selection on kanamycin TM plates allowed the isolation of *TTC0354::pK18* mutants, in which only a C‐terminal deletion form of the protein lacking 121 amino acid could be produced.

## Conflict of interest

None declared.

## Supporting information


**Table S1.** Strains used in this work classified according to their genotype.
**Table S2.** Oligonucleotides used in this work classified according to their purpose.
**Fig. S1.** (A) Multiple sequence alignment by Clustal W of TtSmtB with SmtB/ArsR members. Sequences of the protein used and percentages of identity are: ArsR of T. thermophilus SG0.5JP17‐16, 87%; ArsR of *T. oshimai* JL‐2, 81%; SmtB of *T. scotoductus* SA‐01, 46%; CadC of Clostridium perfrigens, 34%; ZiaR of Synechocystis PCC6803/KAZUSA, 46%; SmtB of Synechococcus PCC7942, 50% and ArsR of *E. coli*, 41%. The ELCVCD motif is highlighted by a red box. HTH domain is indicated. Cys 10 is indicated by a green arrow. Red arrows indicate conserved Cys 62 and Cys 64. Blue arrows indicate residues putatively involved in ligand binding. The secondary structure elements of TtSmtB are depicted above the sequences. (B) Structural model predicted for TtSmtB dimer. (C) Structural model predicted for TTC0354. Modeling of the structures were made on the basis of amino acid sequence by the software I‐TASSER.
**Fig. S2.** Generation of smtB::kat mutant.
**Fig. S3.** Generation of TTC0354 single recombination mutants.
**Fig. S4.** TtSmtB interaction with target promoter.
