# Supplementary material for: An ArsR/SmtB family member regulates arsenic resistance genes unusually arranged in Thermus thermophilus HB27
Source: Microb Biotechnol. 2017 Jul 11;10(6):1690–701. doi: 10.1111/1751-7915.12761 (PMC5658604; doi:10.1111/1751-7915.12761)
Supplement: Supplementary file 1 — Table S1. Strains used in this work classified according to their genotype. Table S2. Oligonucleotides used in this work classified according to their purpose. Fig. S1. (A) Multiple sequence alignment by Clustal W of TtSmtB with SmtB/ArsR members. Sequences of the protein used and percentages of identity are: ArsR of T. thermophilus SG0.5JP17‐16, 87%; ArsR of T. oshimai JL‐2, 81%; SmtB of T. scotoductus SA‐01, 46%; CadC of Clostridium perfrigens, 34%; ZiaR of Synechocystis PCC6803/KAZUSA, 46%; SmtB of Synechococcus PCC7942, 50% and ArsR of E. coli, 41%. The ELCVCD motif is highlighted by a red box. HTH domain is indicated. Cys 10 is indicated by a green arrow. Red arrows indicate conserved Cys 62 and Cys 64. Blue arrows indicate residues putatively involved in ligand binding. The secondary structure elements of TtSmtB are depicted above the sequences. (B) Structural model predicted for TtSmtB dimer. (C) Structural model predicted for TTC0354. Modeling of the structures were made on the basis of amino acid sequence by the software I‐TASSER. Fig. S2. Generation of smtB::kat mutant. Fig. S3. Generation of TTC0354 single recombination mutants. Fig. S4. TtSmtB interaction with target promoter. [file MBT2-10-1690-s001.pdf]

Table S1. Strains used in this work classified according to their genotype.

Strain	Genotype	Source
T. thermophilus HB27	Wild type	DSMZ
T. thermophilus ΔsmtB::*kat*	*T. thermophilus* HB27 deletion mutant of the *TtsmtB* gene, Kan^r	This study
T. thermophilus TTC0354::pK18	*T. thermophilus* HB27 insertion mutant defective in *TTC0354,* Kan^r	This study
E. coli TOP10F'	F'{ $lacI^q$Tn10 (Tet^R)} mcrAΔ(mrr-hsdRMSmcrBC) ϕ80ΔlacZΔM15 Δ lacX74 recA1 deoR araD139 Δ(ara-leu) 7697galU galKrpsL(Str^R) endA1 nupG	Invitrogen
E. coli BL21-Codon Plus (DE3) RIL	F– ompThsdS(r_B– m_B–) dcm+ Tet^r gal λ (DE3) endAHte [argUileYleuWCam^r]	Stratagene

Table S2. Oligonucleotides used in this work classified according to their purpose.

**Primer names**	**Target region**	**Sequences** (the restriction sites are underlined) 5'-3'
qRT-PCR		
0354realfw	*TTC0354*	GACCTCTGCTTTGGCTTTG
0354realrv	*TTC0354*	CTACCTGCCAACTCCTCCA
16Sthfw	*Tt16S*	TAGTCCACGCCCTAAACGAT
16Sthrv	*Tt16S*	CCTTTGAGTTTCAGCCTTGC
smtBrealfw	*TtsmtB*	GAGAGGGTGGTCAAGGAG
0353rv	*TtsmtB*	TCGCAGACGCAAAGCTCC
ArsCrealfw	*TtarsC*	GGAAACCCCTGGAGGAGTG
ArsCrealrv	*TtarsC*	TCGTCGCTGGGAAGCCTTC
Construction of *T.thermophilus* Δ*smtB*::*kat and T.thermophilus* Δ*TTC0354*		
UP fw SmtB EcoRI	arm UP	AAGAATTCTGGAGGATGGAGGGC
NewUPrvSmtBXbaI	arm UP	AAATCTAGAGCAGACCGCCCTAC
NewDWfwSmtBXbaI	arm DW	AAATCTAGATACTACCGCCTCGCC
DW rv SmtB HindIII	arm DW	AAAAAGCTTAAGGCATCCACCACC
0351promfw	0351prom	GAAGCTTGAAGGGGGCTC
0351promrv	0351prom	GCGAGCCACAATACC
0354Eco	0354 internal fw	AAAGAATTCCTCGCCTGGGTTTGGGA
0354Hind	0354 internal rv	AAAAAGCTTGTCCACGGGAGAAAGGCCA

Cloning of the *TtsmtB* gene in pET plasmid		
smtBfw	*TtsmtB*	GTCCAAGGAGGAGGAAACATATGCCAAGCGGGG
smtBrv	*TtsmtB*	GCATCATTTGAGCAAGCTTTCAAGTGTTTTCTTCCGC
EMSA		
0353pr(ext) fw	0353prom	CATGGTGGGCATCAACCT
0353pr(ext) rv2	0353prom	CTCCTTGACCACCCTCTCCT
0354footprint fw	0354prom	CGCCTCGCCGACCGGCA
0354footprint rv	0354prom	CCATGCCCTCTACCCGGAAG
ArsCpr fw	ArsCprom	GGTGGCCCAGCTTGAGCG
ArsCpr rv	ArsCprom	AGAGGACGAGGACCCGCAT
Primer extension		
0353pr(ext) rv 2	*TtsmtB*	CTCCTTGACCACCCTCTCCT
0354 rv	*TTC0354*	CTCGCGTAGTTCACCTGGAC
Footprinting		
0354footprint fw	0354 prom	CGCCTCGCCGACCGGCA
0354footprint rv	0354 prom	CCATGCCCTCTACCCGGAAG

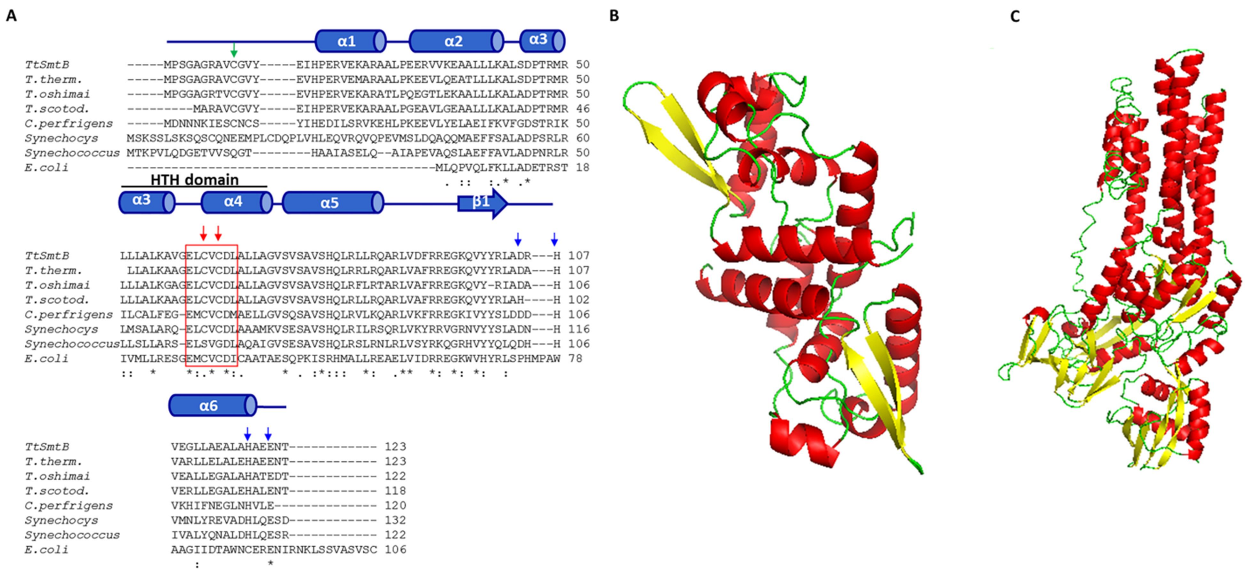

Fig. S1.

A. Multiple sequence alignment by Clustal W of *Tt*SmtB with SmtB/ArsR members. Sequences of the protein used and percentages of identity are: ArsR of *T. thermophilus* SG0.5JP17-16, 87%; ArsR of *T. oshimai* JL-2, 81%; SmtB of *T. scotoductus* SA-01, 46%; CadC of *Clostridium perfrigens*, 34%; ZiaR of *Synechocystis* PCC6803/KAZUSA, 46%; SmtB of *Synechococcus* PCC7942, 50% and ArsR of *E. coli,* 41%. The ELCVCD motif is highlighted by a red box. HTH domain is indicated. Cys 10 is indicated by a green arrow. Red arrows indicate conserved Cys 62 and Cys 64. Blue arrows indicate residues putatively involved in ligand binding. The secondary structure elements of *Tt*SmtB are depicted above the sequences.

B. Structural model predicted for *Tt*SmtB dimer.

C. Structural model predicted for TTC0354.

Modeling of the structures were made on the basis of amino acid sequence by the software I-TASSER.

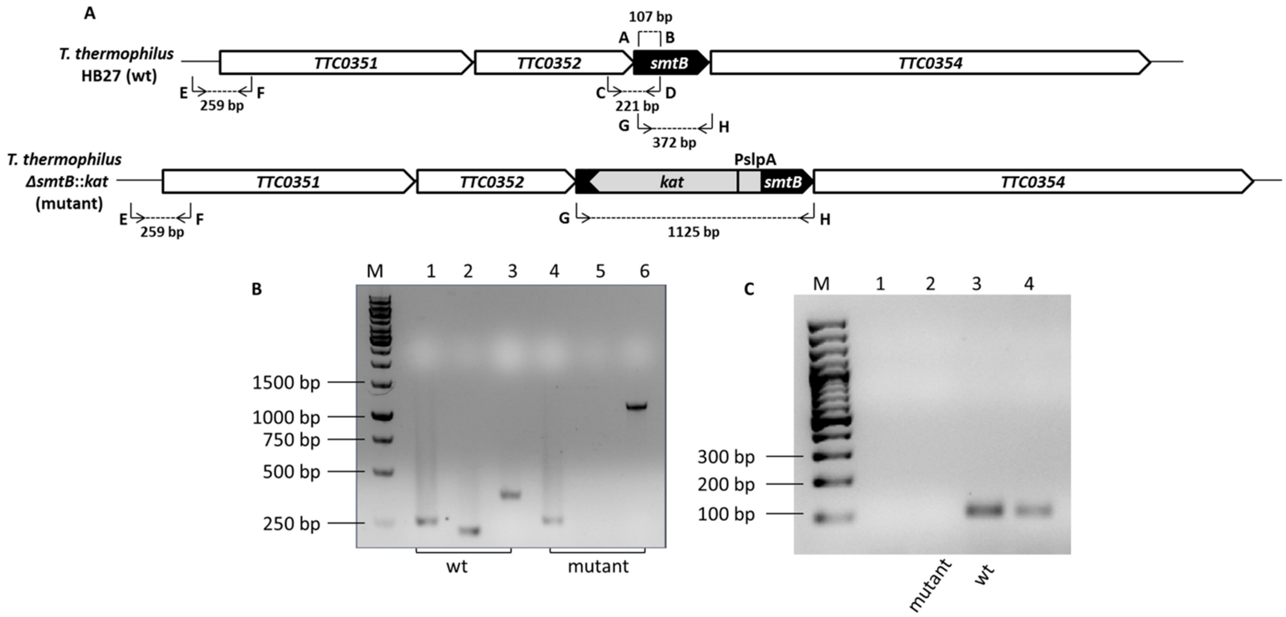

Fig. S2. Generation of *ΔsmtB::kat* mutant.

A. Scheme showing *TtsmtB* and its replacement with the *kat* cassette. Arrows indicate annealing positions and orientation of the primers used to confirm the insertion (Table S2).

B. PCR analysis. Lanes 1 to 3 correspond to DNA of the wild type strain (wt) and lanes 4 to 6 to the mutant. Amplifications were carried out to generate EF (lanes 1,4), CD (lanes 2, 5), and GH fragments (lanes 3,6). Lane M: 1Kb DNA ladder.

C. RT-PCR analysis of the *TtsmtB* gene; lane 1 negative control; lane 2: RT-PCR on *TtsmtB* cDNA from *T. thermophilus ΔsmtB*::*kat*; lane 3: RT-PCR on *TtsmtB* cDNA from the wild type parental strain; lane 4) PCR on genomic DNA of the wild type parental strain. Lane M: 100bp DNA ladder.

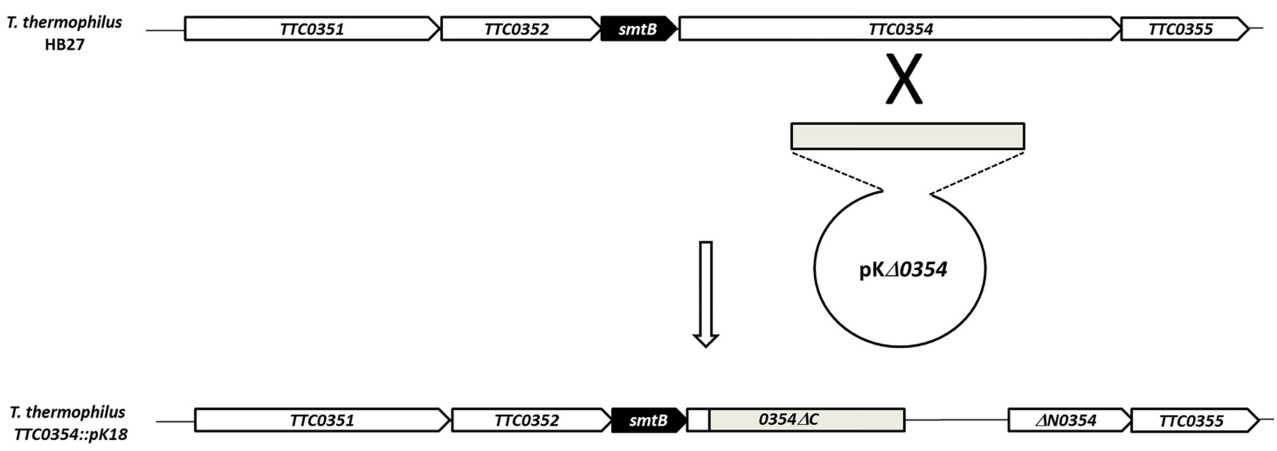

Fig. S3. Generation of *TTC0354* single recombination mutants.

An internal fragment to the targeted gene (*TTC0354*) is cloned into the suicide vector pK18 (containing the kanamycin resistance cassette) to generate pKΔ*0354*. The plasmid is inserted in *T. thermophilus* by single recombination, generating a non functional gene deleted at its C-terminus.

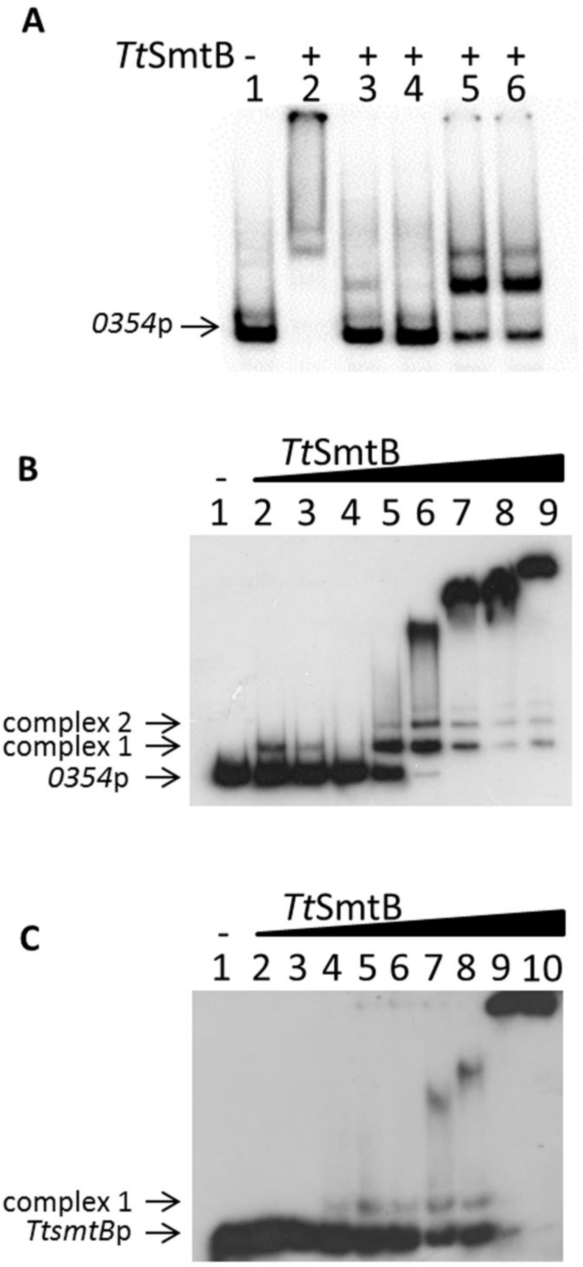

Fig. S4. *Tt*SmtB interaction with target promoter.
A. EMSA competition assays: EMSA with 2.5 μM *Tt*SmtB on *0354*p promoter (1 nM) in the absence (lane 2) or in the presence of unlabeled DNA competitor, either specific (same as the probe, lanes 3,4) or unspecific (lanes 5,6) in a molar ratio of 1:200 and 1:400.B. Binding of *Tt*SmtB to *0354*p: protein concentrations (μM) are: 0 (lane 1), 0.1 (lane 2), 0.2 (lane 3), 0.4 (lane 4), 1.2 (lane 5), 2.5 (lane 6), 5.0 (lane 7), 10.0 (lane 8), 15.0 (lane 9).
C. Binding of *Tt*SmtB to *TtsmtB*p: protein concentrations (μM) are: 0 (lane 1), 0.5 (lane 2), 0.1 (lane 3), 1.5 (lane 4), 2.0 (lane 5), 2.5 (lane 6), 3.7 (lane 7), 5.0 (lane 8), 10.0 (lane 9), 15.0 (lane 10).
